# Genome-wide identification of the OVATE gene family of proteins in soybean and expression profiling under salt stress

**DOI:** 10.3389/fpls.2025.1682513

**Published:** 2025-09-08

**Authors:** Fan Zhan, Yi Wang, Lili Zhang, Yuehua Yu, Zhiyong Ni

**Affiliations:** ^1^ Xinjiang Key Laboratory for Ecological Adaptation and Evolution of Extreme Environment Organisms, College of Life Sciences, Xinjiang Agricultural University, Urumqi, China; ^2^ College of Agronomy, Xinjiang Agricultural University, Urumqi, China

**Keywords:** soybean, OVATE family protein, salt stress, growth, development

## Abstract

OVATE family proteins (OFPs), a class of plant-specific transcription factors, have been increasingly demonstrated to play pivotal roles in multiple aspects of plant growth and development. However, their functional characterization in soybean (*Glycine max*) remains largely unexplored. In this study, we conducted a genome-wide identification of *OFP* genes in soybean, followed by comprehensive analyses, including phylogenetic reconstruction, gene structure characterization, conserved motif and sequence alignment assessments, chromosomal localization, collinearity evaluation, promoter *cis*-acting element profiling, transcriptome-based expression pattern investigation, and quantitative polymerase chain reaction validation. Key findings revealed that the soybean genome harbors 42 *GmOFP* genes, all of which contain the conserved OVATE domain and are distributed unevenly across 19 chromosomes. Most members of this gene family exhibit single-exon architectures, with conserved motif analysis demonstrating that Motif 1 and Motif 2 collectively constitute the OVATE domain. Collinearity analysis indicated that a majority of *GmOFPs* underwent duplication events during evolution. Promoter analysis revealed abundant *cis*-regulatory elements associated with abiotic stress responses, hormonal regulation, light responsiveness, and growth-related processes. Expression profiling revealed that *GmOFP* genes exhibit tissue-specific expression patterns across various soybean organs, with several *GmOFP* genes showing differential responsiveness to salt stress. These findings provide crucial insights into the molecular characteristics and potential biological functions of *GmOFPs*, establishing a theoretical foundation for further investigations into their regulatory mechanisms in soybean growth, development, and stress adaptation.

## 1 Introduction

The OVATE family proteins (OFPs) represent a class of plant-specific transcription factors characterized by a conserved OVATE domain at their C-terminus, comprising approximately 70 amino acids. These proteins, which were initially identified in tomato (*Solanum lycopersicum*), are hydrophilic and contain nuclear localization signals (Liu et al., 2002). Studies have demonstrated that *OFP* family members play regulatory roles in diverse plant developmental processes, including organ morphogenesis, hormone signal transduction, abiotic stress responses, and secondary cell wall formation, highlighting their critical involvement in key physiological functions (Wang et al., 2022; Xia et al., 2023; Ding et al., 2020; Borovsky et al., 2022; Liu et al., 2018).

In plant organ morphogenesis, *OFPs* play pivotal regulatory roles. As a critical factor governing organ development, the *OVATE* gene in tomato, when harboring a premature termination codon mutation, not only transforms spherical fruits to a pear-shaped phenotype but also significantly reduces the floral organ size and diminishes leaf growth scales (Liu et al., 2002). In *Arabidopsis*, *AtOFP1* functions as a cellular morphology regulator by suppressing the transcriptional activity of *AtGA20ox1*, a key gene in gibberellin (GA)biosynthesis, thereby negatively regulating cell elongation (Wang et al., 2007). The upregulation of the rice (*Oryza sativa*) *OsOFP2* gene induces dwarfism, disrupts leaf development, causes grain morphological deformities, and reorganizes vascular bundle architecture in stems through its influence on the cellular polarity growth regulatory network (Schmitz et al., 2015). Similarly, knockdown of *OsOFP6* leads to plant dwarfing, altered grain shape, and shortened lateral roots (Ma et al., 2017). In *Arabidopsis*, KNAT7 collaborates with AtOFP1 and AtOFP4 via a protein interaction network to increase the transcriptional repression of target genes synergistically, a molecular mechanism critically involved in regulating secondary cell wall biosynthesis (Li et al., 2011). Additionally, OVATE family proteins coordinate with TONNEAU1 to modulate fruit morphology by influencing cell division patterns (Wu et al., 2018). Although these studies span diverse species and tissues, they collectively support a unifying conclusion: *OFPs* primarily regulate plant growth and development through transcriptional repression mechanisms.

*OFPs* play crucial roles in multiple plant hormone signaling pathways and auxin regulation. In rice, *OsOFP6* has been shown to potentially suppress the expression of auxin signaling-related genes, thereby modulating this pathway. Notably, RNA interference-mediated knockdown of this gene resulted in a significantly stronger regulatory effect on downstream target genes than did the phenotypic changes induced by its overexpression. This observation further supports the biological function of *OsOFP6* as a key repressor in the auxin signaling network, suggesting that this protein may finely regulate auxin homeostasis in plants through negative feedback mechanisms (Ma et al., 2017). During lettuce (*Lactuca sativa*) development, the *LsOFP6* gene exhibits marked spatiotemporal expression specificity, with its expression peaking at the critical transition stage from vegetative to reproductive growth. Recent studies revealed that the *LsKN1-LsOFP6* molecular module regulatory network achieves precise control of bolting time by bidirectionally modulating both the biosynthetic pathway and signaling response system of GA (Qi et al., 2025). Research on the brassinosteroid (BR) signaling pathway has revealed more complex regulatory networks involving *OFPs*. Rice OFPs have been demonstrated to participate in BR signal transduction and influence grain size. Specifically, FBX206 interacts with OsOFP8 (a positive regulator) and OsOFP19 (a negative regulator) in the BR signaling pathway, forming a coordinated regulatory pathway that ultimately determines grain size and yield (Sun et al., 2024). Additionally, studies have shown that OsOFP19, OSH1, and DLT can form a functional complex critical for plant growth and development. This complex regulates BR signaling and determines cell division patterns, with DLT notably suppressing the individual activities of both OsOFP19 and OSH1 and their synergistic regulatory effects on gene expression (Yang et al., 2018). These findings collectively demonstrate that *OFPs* execute intricate and precise regulatory functions across diverse developmental stages and tissues by participating in multiple phytohormone signaling pathways. They likely act as molecular hubs to integrate different hormone signals, thereby coordinating plant growth and developmental processes.

*OFP* family members play crucial regulatory roles in plant responses to abiotic stresses. Studies have demonstrated that these proteins participate in plant adaptation to drought, salinity, and other adverse conditions through diverse molecular mechanisms. In rice, drought resistance is positively correlated with *OsOFP6* expression levels. The *OFP6*-overexpressing lines presented a relatively low rate of water loss and reduced H_2_O_2_ accumulation, whereas the *OsOFP6*-knockdown plants presented the opposite phenotypes, suggesting that *OsOFP6* may increase drought avoidance and tolerance by regulating oxidative stress responses (Ma et al., 2017). In woody plants, *Populus trichocarpa PtOFP1* was identified as a transcriptional repressor. Heterologous overexpression of *PtOFP1* in *Arabidopsis* enhanced tolerance to PEG-mediated drought stress at the seedling stage and increased survival rates at the mature stage, indicating that *PtOFP1* may improve drought resistance through conserved regulatory mechanisms (Wang et al., 2021). Moreover, OFPs can interact with other transcription factors to regulate stress responses in a coordinated manner. For example, PpOFP1 interacts with PpZFHD1 to increase salt tolerance synergistically in transgenic tomato plants (Tan et al., 2021). In *Arabidopsis*, *AtOFP8* modulates cuticular wax biosynthesis by regulating the expression of wax-related genes, potentially influencing leaf surface wax deposition to reduce transpirational water loss and improve drought resistance (Tang et al., 2018). Taken together, *OFPs* play multifaceted roles in plant responses to abiotic stress, including the regulation of oxidative stress, the modulation of water metabolism, the enhancement of salt tolerance, and the modification of epidermal structures.

Soybean, a globally vital legume crop, has substantial nutritional and economic value. However, its productivity is severely reduced due to the detrimental impacts of salt stress. *OFP* proteins play significant roles in mediating plant responses to salt stress. However, the function of the *OFP* gene in the response of soybean to salt stress has not yet been elucidated. Therefore, we conducted a genome-wide analysis of the soybean *OFP* gene family and systematically investigated its expression patterns under salt stress. The research included family member identification, chromosomal localization, phylogenetic tree construction, gene structure analysis, collinearity relationship assessment, and promoter *cis*-acting element characterization. Furthermore, we analyzed the tissue-specific expression profiles of the soybean *OFP* genes using genomic databases and publicly available RNA-seq data, with a particular focus on their expression dynamics under salt stress. The expression patterns of the soybean *OFP* genes were further validated through experimental approaches. In summary, our findings provide valuable insights and theoretical foundations for elucidating the functional roles of soybean *OFP* genes and their potential involvement in salt stress responses.

## 2 Materials and methods

### 2.1 Plant materials

The plant material used in this study was the soybean cultivar Williams 82. The seedlings were cultivated under controlled environmental conditions, with a temperature regimen of 26°C and a 14-hour light/10-hour dark photoperiod. At the cotyledon (VC) growth stage (approximately 14 days post-germination, which is characterized by full expansion of two unifoliate leaves), the plants were subjected to salt stress treatment with 250 mM NaCl. Root tissues were sampled at 0, 6, 12, and 24 hours post-treatment initiation, with three biological replicates collected at each time point. Harvested samples were immediately flash-frozen in liquid nitrogen and stored at −80°C for subsequent analyses.

### 2.2 Screening and identification of the *OFP* gene family in soybean

The soybean genome (Wm82.gnm4) sequence and corresponding annotation files were retrieved from the Phytozome database (*G. max* Wm82.a4.v1: Phytozome; accessed 15 February 2025). The hidden Markov model (HMM) profile of the OVATE domain (PF04844) was downloaded through the Pfam plugin in the InterPro database (https://www.ebi.ac.uk/interpro/entry/pfam/; accessed 15 February 2025) (Bateman et al., 2002). Genome-wide screening for OVATE domain-containing genes was performed using the HMM search plugin in TBtools software (https://github.com/CJ-Chen/TBtools/releases; accessed 15 February 2025) against the soybean proteome (Chen et al., 2023; Zhang et al., 2021). Candidate genes were subsequently validated through domain architecture analysis using the NCBI Conserved Domains Database (CDD; https://www.ncbi.nlm.nih.gov/Structure/cdd/wrpsb.cgi) and SMART database (https://smart.embl.de/; both accessed 17 February 2025) (Letunic et al., 2002). Proteins lacking conserved OVATE-associated domains were manually excluded. Comprehensive analysis of protein physicochemical properties of the identified *GmOFP* family members, including amino acid number, molecular weight (MW), isoelectric point (pI), instability index, aliphatic index, and grand average of hydropathicity (GRAVY), was conducted using TBtools (Fang et al., 2021).

### 2.3 Phylogenetic analysis of the *OFP* gene family in soybean

The protein sequences of the *OFP* gene family in *Arabidopsis* (retrieved from https://www.arabidopsis.org/, accessed on February 20, 2025) and *Oryza sativa* (obtained from https://rapdb.dna.affrc.go.jp/, accessed on February 20, 2025) were acquired using the methodology described in Section 2.2. These sequences were subsequently aligned through multisequence alignment performed with ClustalW software. An integrated dataset comprising *OFP* family members from *Arabidopsis*, rice, and soybean was then compiled for phylogenetic analysis. A neighbor-joining (NJ) phylogenetic tree was constructed using MEGA-7.0 software with 1000 bootstrap replicates to assess node reliability (Kumar et al., 2018; Belamkar et al., 2014). Final tree visualization and annotation were conducted through the iTOL online platform (https://itol.embl.de/, accessed on February 23, 2025) to enhance topological clarity and graphical presentation (Jiang et al., 2024).

### 2.4 Comprehensive analysis of gene structure, conserved motifs, and sequence alignment in the *OFP* gene family of soybean

The gene structures of the soybean *OFP* gene family members were analyzed and visualized using TBtools software on the basis of their DNA sequences, protein sequences, and soybean genome annotation information (Zhang et al., 2016). Conserved motifs in the soybean *OFP* gene family were predicted through the MEME suite (https://meme-suite.org/meme/, accessed on February 27, 2025) and subsequently visualized using TBtools software (Bailey et al., 2009). Multiple sequence alignment of the soybean *OFP* gene family was performed using Jalview software (https://www.jalview.org/, accessed on March 2, 2025) to investigate sequence conservation patterns (Waterhouse et al., 2009).

### 2.5 Chromosomal localization and collinearity analysis of the *GmOFP* gene

The chromosomal location of the *GmOFP* gene was visualized using the Gene Location Visualize plugin within the GTF/GFF annotation module of TBtools software (Chen et al., 2023). Furthermore, intra- and interspecies syntenic relationships of the soybean *OFP* gene family were analyzed using the MCScanX plugin in TBtools, with *Arabidopsis* and *Oryza sativa* selected as comparative species (Wang et al., 2012).

### 2.6 Comprehensive analysis of *cis-*acting elements in the promoter regions of the *GmOFP* gene family in soybean

The upstream 2000 bp promoter sequences of the soybean *OFP* family genes were extracted using TBtools software. The extracted promoter sequence files were subsequently submitted to the PlantCARE web server (https://bioinformatics.psb.ugent.be/webtools/plantcare/html/; accessed on 7 March 2025) for systematic prediction of *cis*-acting regulatory elements. Lastly, visualization and bioinformatics analysis of the identified *cis*-regulatory elements were performed using TBtools software (Wang et al., 2024; Chen et al., 2020).

### 2.7 Transcriptome database-based expression profiling analysis of the *GmOFP* gene in soybean

The RNA-seq data for root tissues under salt stress in the soybean cultivar Williams 82 were retrieved from the NCBI Gene Expression Omnibus (GEO) database (https://www.ncbi.nlm.nih.gov/geo/; accessed on March 7, 2025). Concurrently, tissue-specific expression patterns of *GmOFP* genes were acquired from the SoyBase genomic resource (https://www.soybase.org/; accessed on March 9, 2025) (Libault et al., 2010; Razzaq et al., 2023). Visualization of *GmOFP* gene expression profiles was performed through heat map generation using the HeatMap Illustrator module within the TBtools bioinformatics platform (Luo et al., 2022).

### 2.8 RNA extraction and quantitative polymerase chain reaction validation

Total RNA was isolated from soybean root tissues subjected to salt stress at 0, 6, 12, and 24 hours using a Plant Total RNA Extraction Kit (Tiangen Biotech (Beijing) Co., Ltd., Beijing, China), with three biological replicates collected per time point. Genomic DNA removal and first-strand cDNA synthesis were performed according to the manufacturer’s protocol using the EasyScript^®^ One-Step gDNA Removal and cDNA Synthesis SuperMix (TransGen Biotech (Beijing) Co., Ltd., Beijing, China). qPCR analysis was conducted on a LightCycler 96 system with PerfectStart^®^ Green qPCR SuperMix (TransGen Biotech (Beijing) Co., Ltd., Beijing, China) in a 20 μL reaction volume. The soybean *GmCYP2* gene served as an internal reference for normalizing the expression levels of selected *GmOFP* genes under salt stress. Relative gene expression levels were calculated using the 2^−ΔΔCt^ method, with data processing performed in Excel 2019 (Cheng et al., 2024). The sequences of primers used for the qPCR analysis are detailed in **Supplementary Table S1**.

### 2.9 Statistical analysis

Statistical analyses, including Student’s t test and one-way ANOVA, were con-ducted using GraphPad Prism 10.1 (GraphPad Software), with column graphs subsequently generated to illustrate the relative expression levels.

## 3 Results

### 3.1 Screening and identification of the *OFP* gene family in soybean

In this study, we systematically identified genes encoding the OVATE domain in the soybean genome through initial screening followed by validation using the NCBI-CDD and the SMART database. Genes not encoding the OVATE domain were manually excluded, and redundant sequences were removed through rigorous filtering. This process yielded 42 non-redundant *OFP* gene family members in the soybean genome. These genes were designated *GmOFP1* through *GmOFP42* according to their chromosomal localization; comprehensive gene information is presented in **Supplementary Table S2**. Physicochemical characterization revealed that all the encoded proteins except *GmOFP2* exhibited hydrophilic properties. The amino acid lengths of these proteins ranged from 152 to 414 residues. Further analyses of critical biochemical parameters, including the MW, pI, instability index, and aliphatic index, were conducted, and the complete data are summarized in **Supplementary Table S2**. Notably, the observed variations in these parameters suggest functional diversity within the *OFP* family, which may be associated with distinct biological roles in soybean development.

### 3.2 Phylogenetic analysis of the *OFP* gene family in soybean

**Figure 1:**
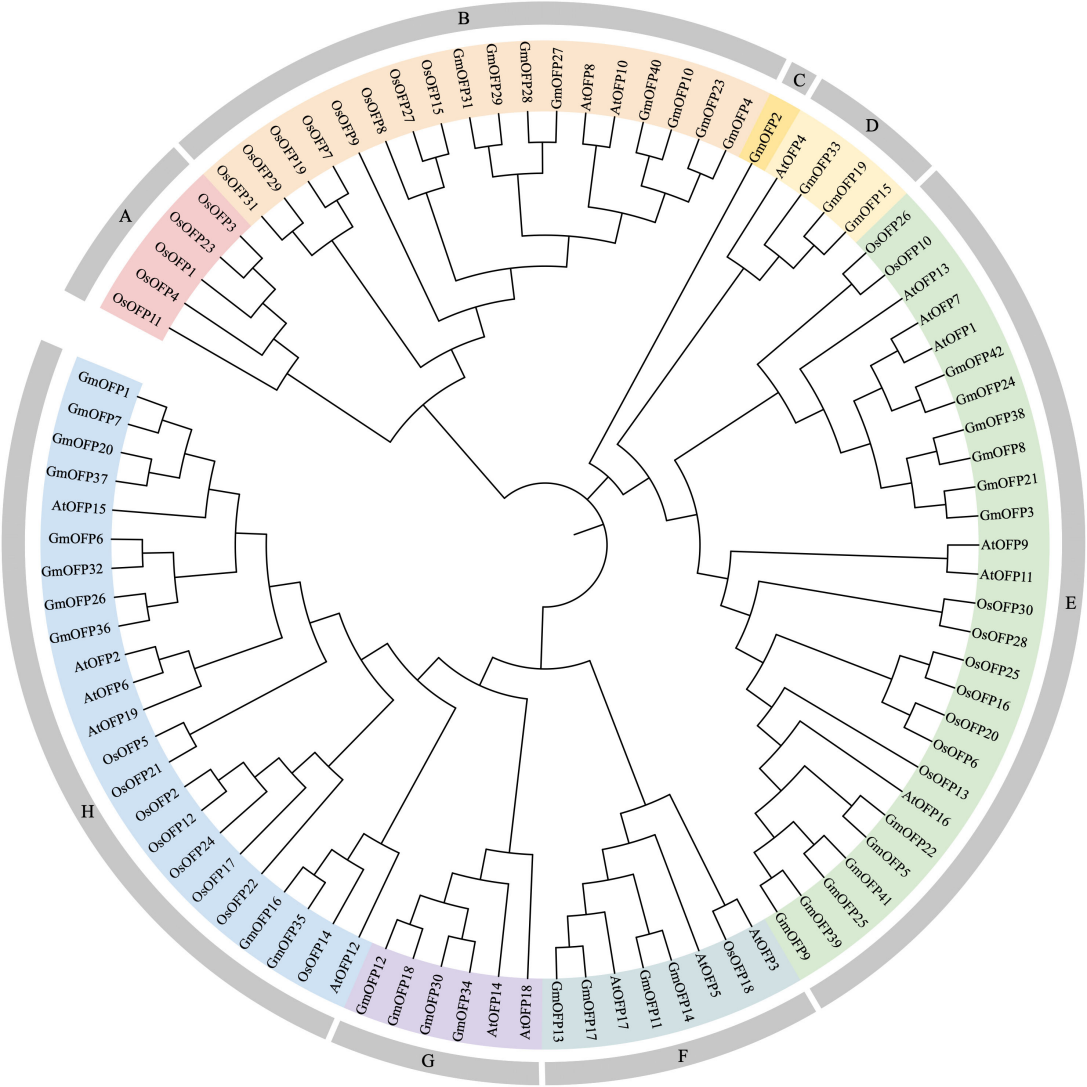
Phylogenetic trees of *OFP* gene family members in soybean, *Arabidopsis*, and rice. Phylogenetic analysis of *OFP* gene family members in soybean (42), *Arabidopsis* (19), and rice (31) was conducted using MEGA 7.0 software. Different uppercase letters represent different subfamilies.

To investigate the phylogenetic relationships of the *OFP* gene family in soybean, we retrieved *OFP* gene sequences from *Arabidopsis* (19 members) and *Oryza sativa* (31 members) and constructed an evolutionary tree using the NJ method (**Figure 1**). Phylogenetic analysis revealed that these OFP proteins clustered into eight distinct subfamilies, designated Groups A to H. Among the 42 soybean *OFP* genes, the members were distributed across seven subfamilies: Group B contained eight members, Group C one, Group D three, Group E twelve, Group F four, Group G four, and Group H ten. Notably, soybean *OFP* genes co-clustered with their *Arabidopsis* and rice orthologs in varying proportions within specific subfamilies, suggesting conserved evolutionary trajectories and functional diversification across these species. This shared phylogenetic topology implies potential homology and parallel evolutionary processes among *OFP* genes in soybean, *Arabidopsis*, and rice.

### 3.3 Analysis of the gene structure, conserved motifs and sequence alignment of the soybean *OFP* gene family

**Figure 2:**
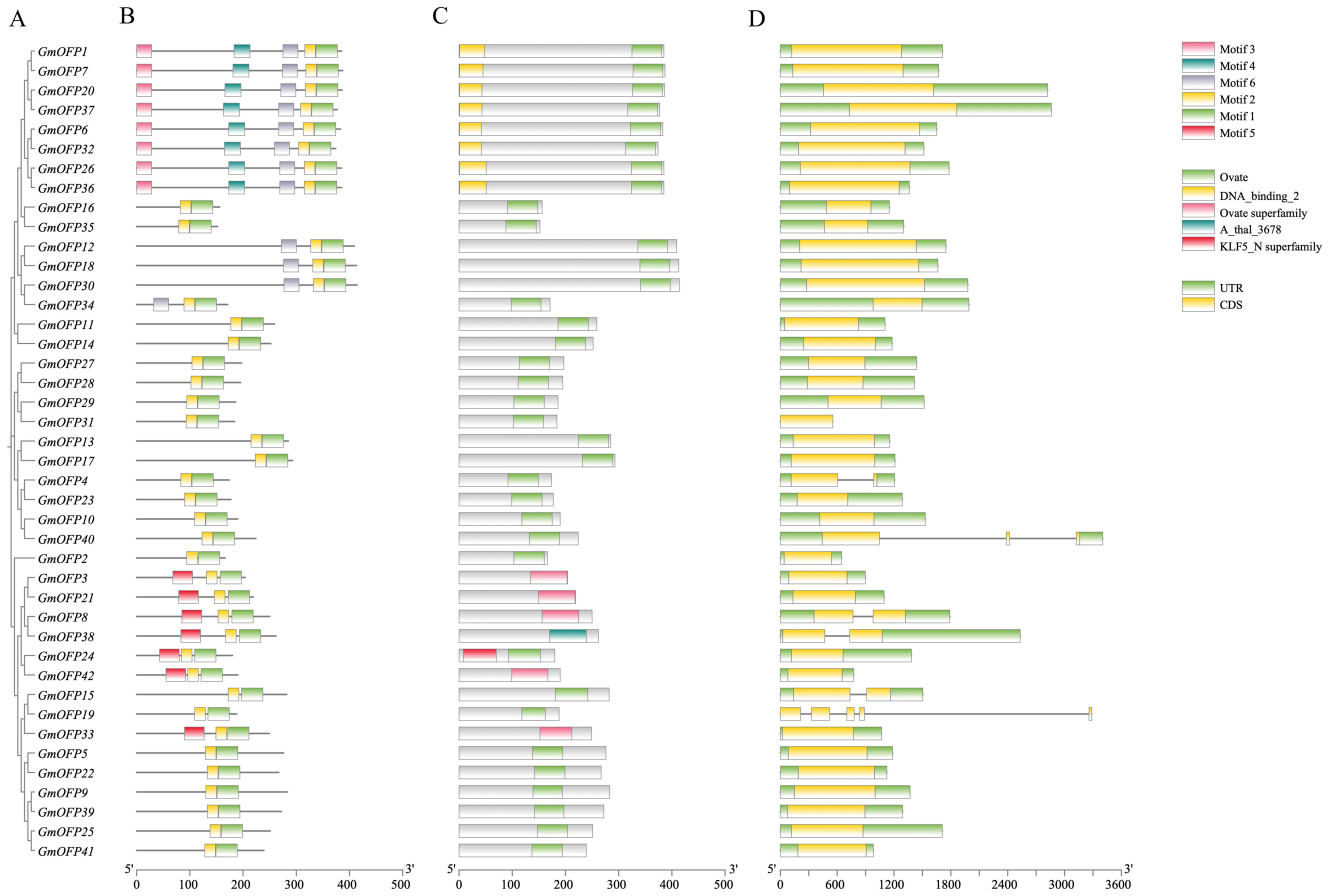
Conserved motif and gene structure analysis of the soybean *OFP* gene family. **(A)** Phylogenetic tree of the soybean *OFP* gene family. **(B)** Schematic diagram of the structural distribution of the conserved motifs of the soybean *OFP* gene, in which different colors represent different conserved motifs. **(C)** Distribution of the OVATE conserved domain of the soybean *OFP* gene. **(D)** Structure of the soybean *OFP* gene, where yellow represents exons and green represents the 5’UTR and 3’UTR.

To elucidate the fundamental architecture of soybean *OFP* gene family members, we conducted a comprehensive series of analyses. Initially, phylogenetic clustering on the basis of gene structure (**Figure 2A**) revealed that members with similar structural organization were grouped into distinct clades. Conserved motif analysis (**Figure 2B**, **Supplementary Figure S1**) revealed six characteristic motifs distributed across all 42 *OFP* family members. Notably, Motif 1 and Motif 2 exhibited universal conservation and were present in all the members with consistent spatial proximity. Gene structure characterization (**Figure 2C**) revealed that all the family members contained the diagnostic OVATE domain (including the A_thal_3678 domain) in their C-terminal regions. Through comparative analysis of motif distribution patterns and frequency, we hypothesize that Motif 1 and Motif 2 collectively constitute the core components of the OVATE domain. Intriguingly, structural examination revealed that a majority of these genes possess single-exon architectures, a feature potentially associated with evolutionary conservation and enhanced regulatory efficiency (**Figure 2D**). Multiple sequence alignment further confirmed the universal presence of OVATE protein domains across all family members, reinforcing their structural conservation within this gene family (**Supplementary Figure S2**).

### 3.4 Chromosomal localization and collinearity analysis of the *GmOFP* gene

**Figure 3:**
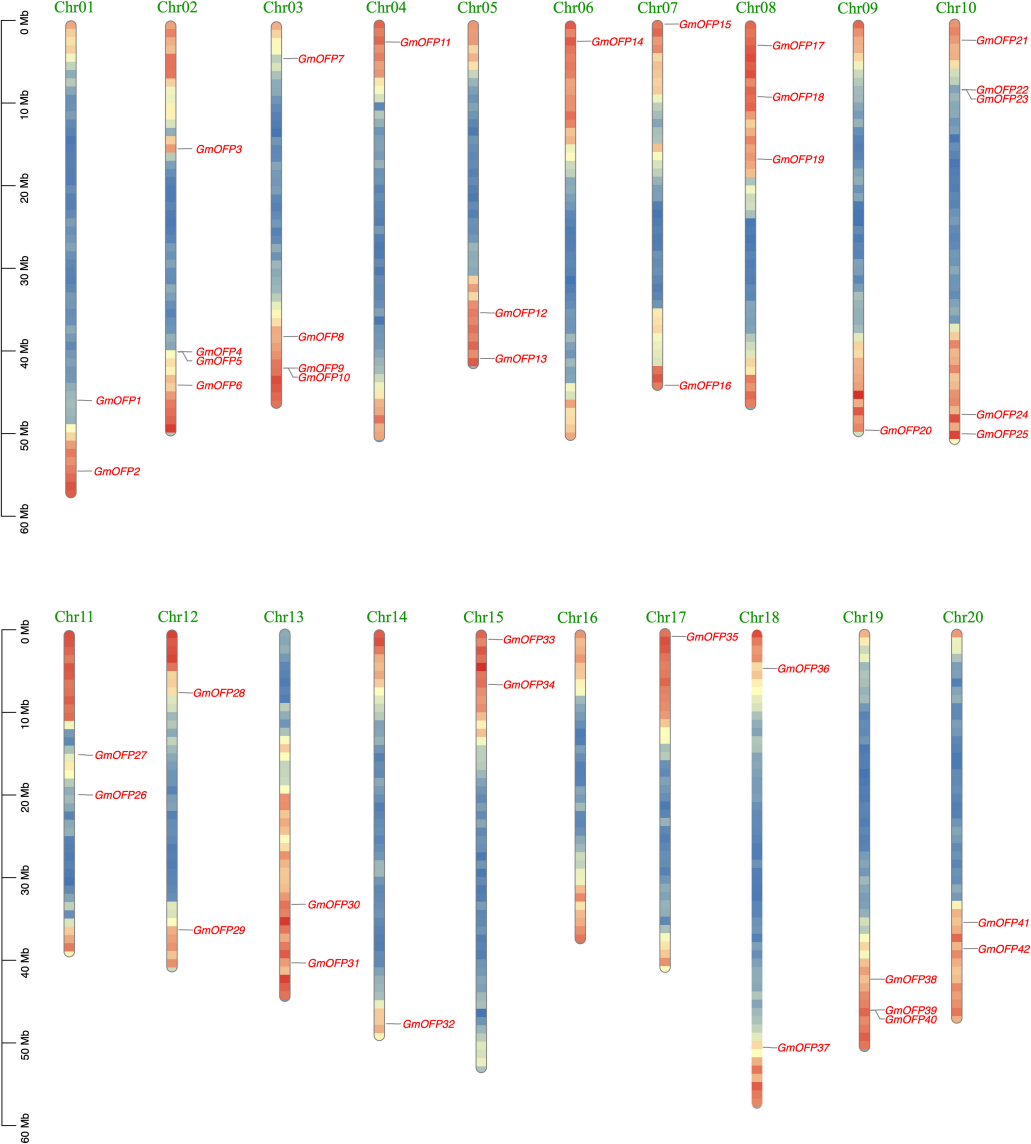
Chromosome localization of the soybean *OFP* gene family.

Through the chromosomal localization of the *OFP* gene in soybean, we found that the 42 members of the soybean *OFP* gene family were unevenly distributed on 19 chromosomes (**Figure 3**), and most of the genes were located on both sides of the head and tail of the chromosomes (head or tail). Among them, the *GmOFP* gene is not present on chromosome 16. One *GmOFP* gene is located on chromosomes 4, 6, 9, 14 and 17. There are two *GmOFP* genes on chromosomes 1, 5, 7, 11, 12, 13, 15, 18 and 20. There are three *GmOFP* genes on chromosomes 8 and 19. There are four *GmOFP* genes on chromosomes 2 and 3. There are five *GmOFP* genes on chromosome 10.

**Figure 4:**
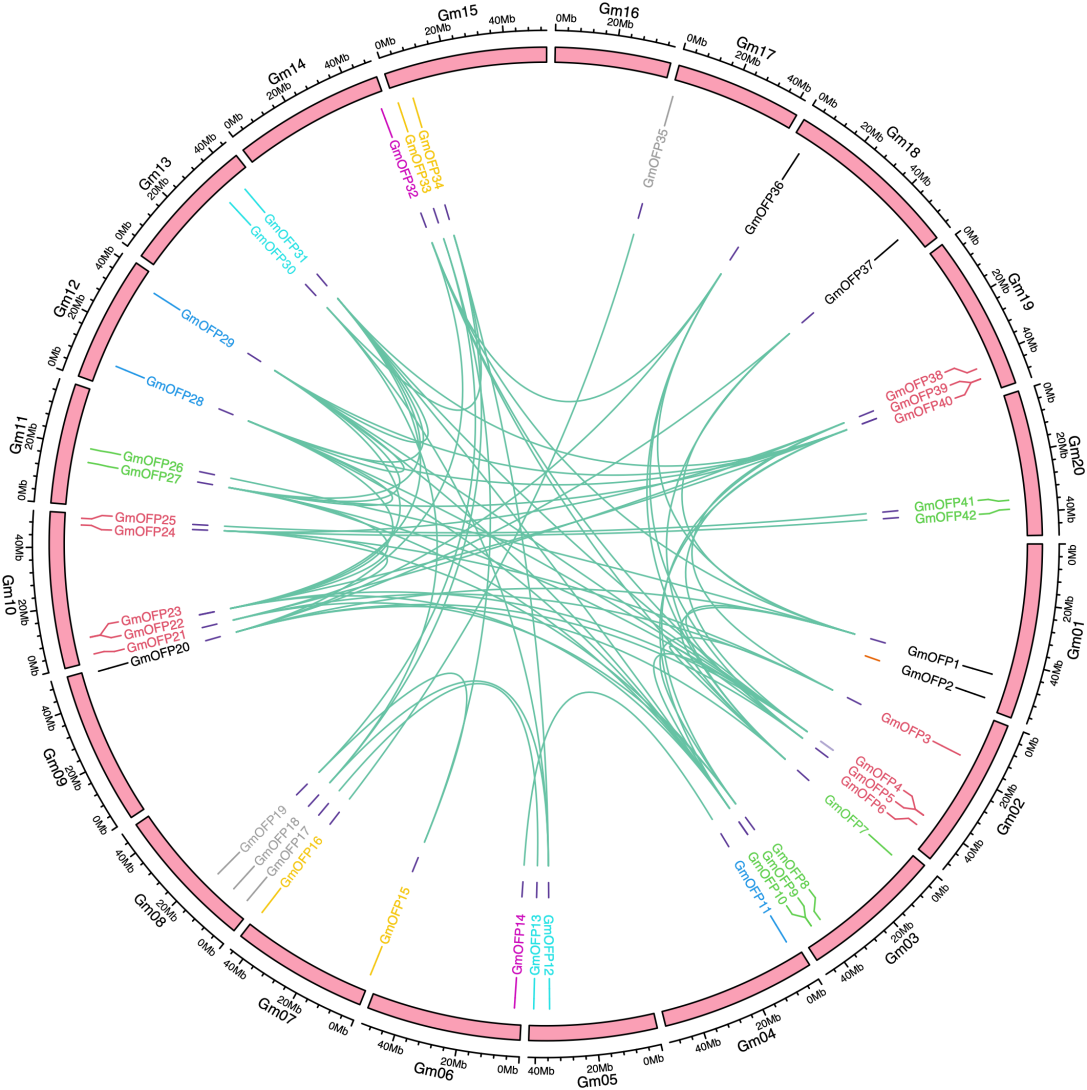
Intraspecies collinearity analysis of the soybean *OFP* gene family. The pink squares represent chromosomes, and the gene pairs with fragment repetitions are connected by green lines.

**Figure 5:**
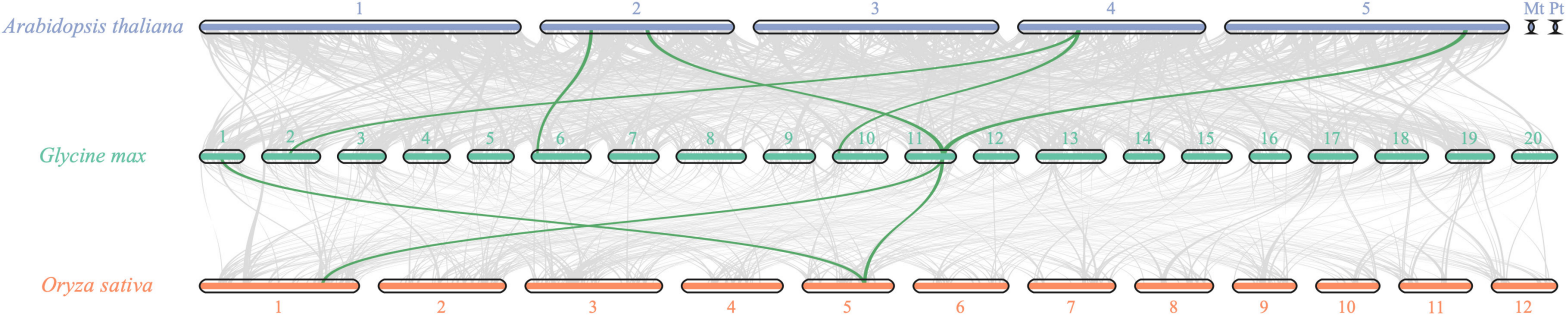
Collinearity analysis of *OFP* gene family members in soybean, *Arabidopsis*, and rice. The bar squares represent chromosomes (purple bar squares represent *Arabidopsis* chromosomes, green bar squares represent soybean chromosomes, and orange bar squares represent rice chromosomes), and gene pairs with fragment duplications are connected by green lines.

To investigate the evolutionary dynamics and gene duplication relationships among members of the soybean *OFP* gene family, we conducted intraspecies collinearity analysis of *OFP* family members in *Glycine max* (**Figure 4**). The results revealed numerous homologous gene pairs among the 42 identified *OFP* family members, demonstrating the conserved evolutionary characteristics of this gene family and indicating multiple gene duplication events during its evolutionary history. Furthermore, cross-species collinearity analyses between soybean and divergent species (*Oryza sativa* and *Arabidopsis*) were performed (**Figure 5**). These interspecific comparisons revealed conserved syntenic relationships and evolutionary conservation of duplication events across different lineages, suggesting that these collinear gene pairs may have originated from ancestral genomic regions prior to species divergence. The persistence of syntenic relationships across evolutionary timescales provides evidence for the functional conservation and ancient origins of *OFP* gene family expansion mechanisms in plants.

### 3.5 Analysis of *cis*-acting elements in the promoter of the *GmOFP* gene family

**Figure 6:**
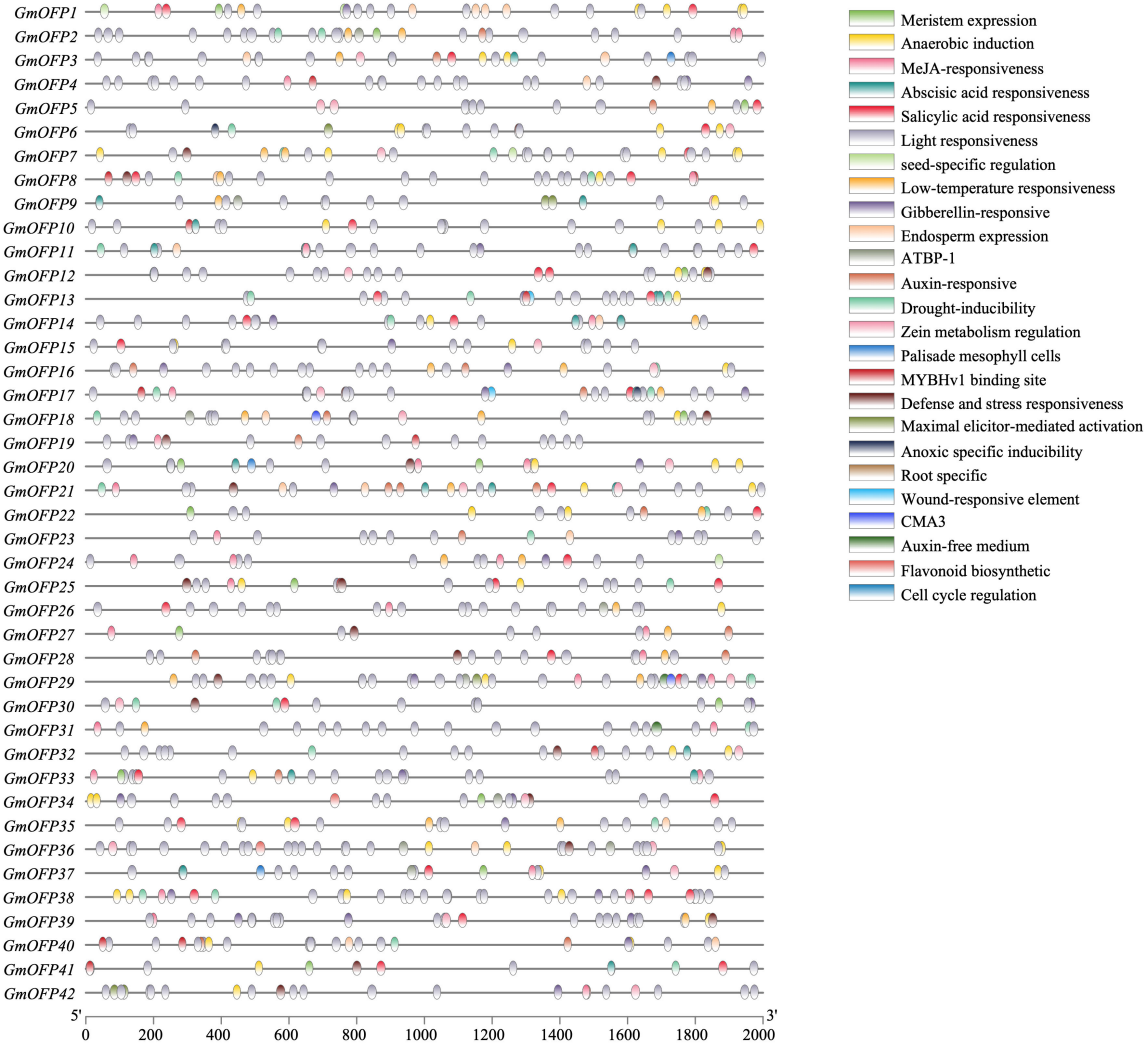
Transcriptional regulatory elements of the promoter region of soybean *OFP* genes. There are 25 types of transcriptional regulatory elements in the soybean *OFP* gene family. Different colors represent different types of regulatory elements.

To investigate the regulation of soybean *OFP* gene expression, we extracted the 2000-bp promoter sequences upstream of its members and performed *cis*-acting element prediction using the PlantCARE database. Bioinformatics analysis revealed 25 distinct *cis*-regulatory elements associated with abiotic stress responses, hormonal regulation, light responsiveness, and plant growth/development (**Figure 6**). The coordinated presence of these diverse regulatory motifs reveals the molecular basis for functional diversification within the *OFP* gene family. Furthermore, the differential *cis*-regulatory element combinations among family members suggest potential mechanisms for precise transcriptional regulation through collaborative interactions with other gene family products during specific developmental stages or under particular environmental conditions. These findings collectively demonstrate that the soybean *OFP* gene family plays crucial regulatory roles in plant growth and developmental processes.

### 3.6 Transcriptome database-based expression profiling analysis of the *GmOFP* gene in soybean

**Figure 7:**
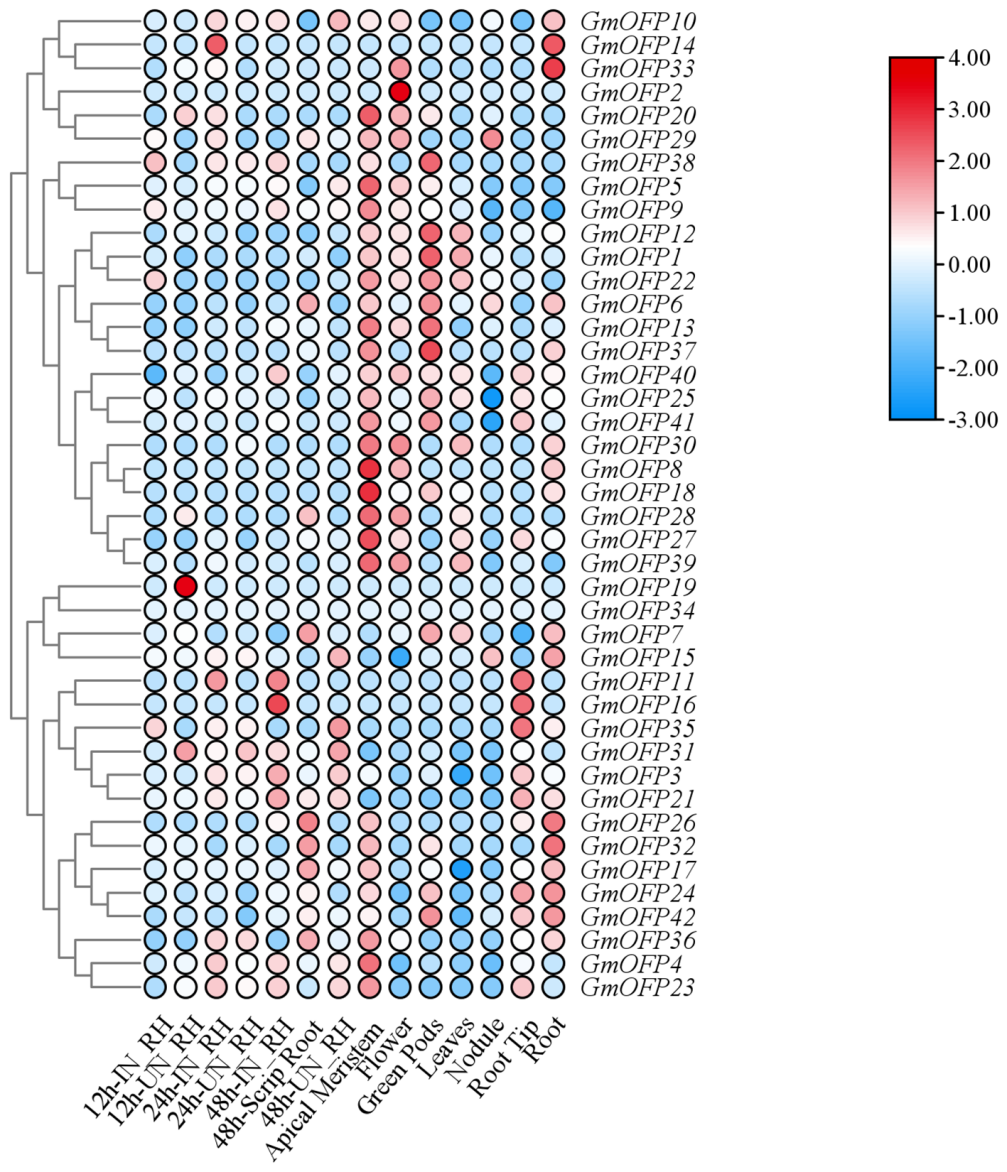
Expression patterns of the soybean *OFP* gene in inoculated rhizobia and different tissues. Cluster analysis was conducted on the expression patterns of 42 *GmOFP* genes in soybeans before and after rhizobium inoculation and in different tissues. Blue indicates low expression levels, and red indicates high expression levels.

Through integrative analysis of RNA-seq data and the soybean genomic database, we systematically investigated the expression patterns of the *Glycine max OFP* gene family across diverse tissues and under rhizobial inoculation and salt stress conditions. Comparative expression profiling of *GmOFP* members between the rhizobium-inoculated (IN_RH) and uninoculated (UN_RH) treatments at 12, 24, and 48 hours post-inoculation revealed distinct responsiveness patterns. Multiple *GmOFP* genes, including *GmOFP4*, *GmOFP23*, *GmOFP31*, and *GmOFP35*, exhibited transcriptional responsiveness to rhizobial symbiosis, whereas a subset (e.g., *GmOFP2*, *GmOFP8*, *GmOFP18*, and *GmOFP34*) maintained constitutive expression unaffected by inoculation (**Figure 7**). Tissue-specific expression profiling demonstrated spatial regulation of *GmOFP* members: increased transcript accumulation was observed for *GmOFP8*, *GmOFP18*, *GmOFP27*, and *GmOFP20* in apical meristem tissues, whereas *GmOFP14*, *GmOFP26*, *GmOFP32*, and *GmOFP33* showed preferential expression in root tissues. Differential expression levels were further detected across reproductive and vegetative organs, with distinct *GmOFP* members displaying modulated transcriptional activity in flowers, green pods, leaves, root nodules, and root tips. These findings collectively suggest both functional diversification and context-dependent regulation occur within the *GmOFP* gene family during developmental processes and biotic interactions.

**Figure 8:**
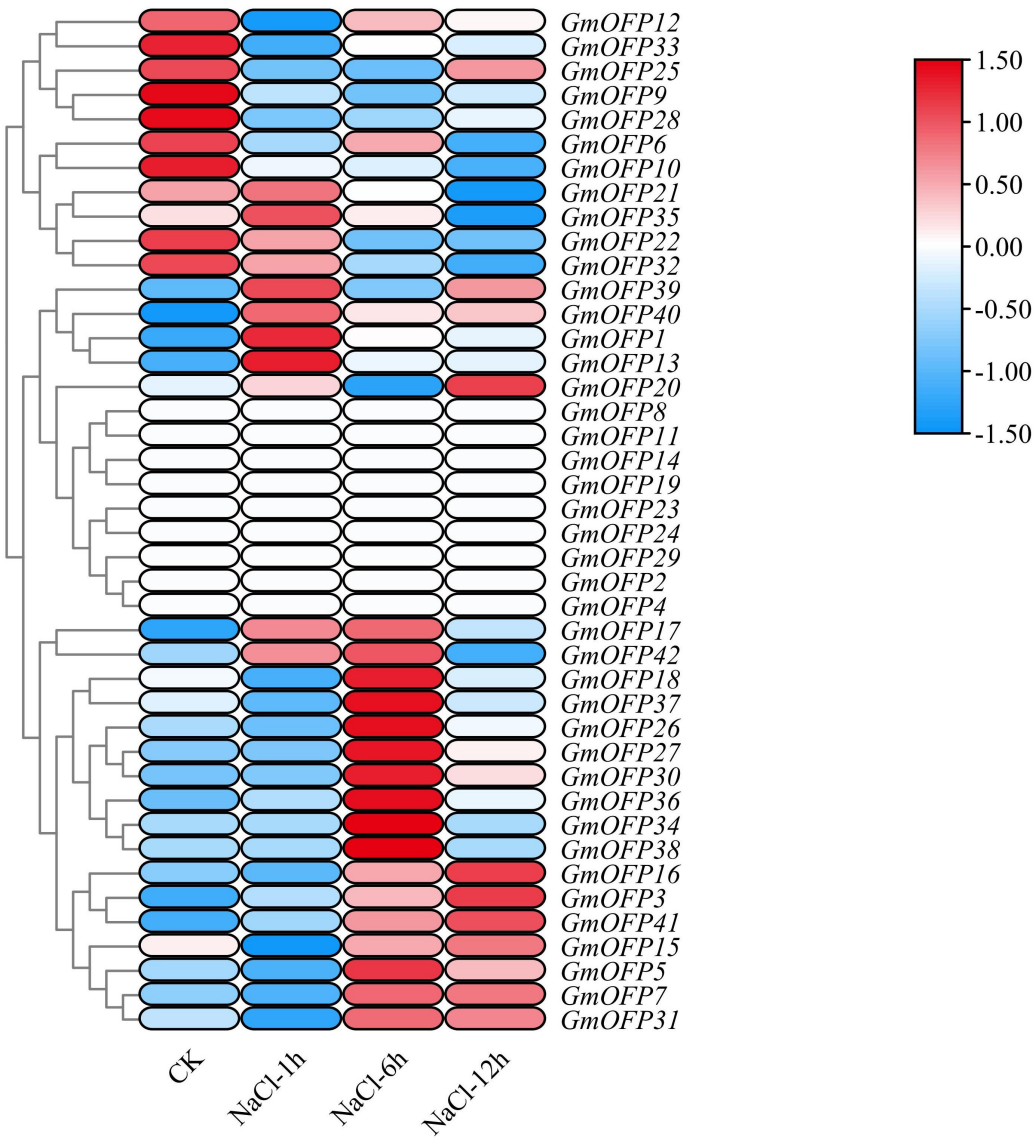
Expression pattern of the *GmOFP* gene in soybeans under salt stress. A cluster analysis was conducted on the expression patterns of 42 *GmOFP* genes in the transcriptome of soybean plants under salt stress. The blue color indicates low expression levels, whereas the red color indicates high expression levels.

In the analysis of expression patterns under salt stress, we observed that a majority of the soybean *GmOFP* genes responded to salt stress (**Figure 8**). Compared with those under the control conditions, the transcript levels of genes such as *GmOFP9*, *GmOFP12*, *GmOFP25*, and *GmOFP33* were downregulated following salt stress treatment. Conversely, the transcript levels of genes such as *GmOFP3*, *GmOFP7*, *GmOFP16*, and *GmOFP41* were markedly upregulated under the same stress conditions. Notably, the expression profiles of genes such as *GmOFP11*, *GmOFP14*, *GmOFP19*, and *GmOFP23* remained unaltered or showed no detectable expression in response to salt stress exposure. These expression patterns indicate that these genes may exhibit functional diversity in regulating developmental processes in soybean under salt stress conditions.

### 3.7 qPCR detection of the *GmOFP* gene in soybean under salt stress

**Figure 9:**
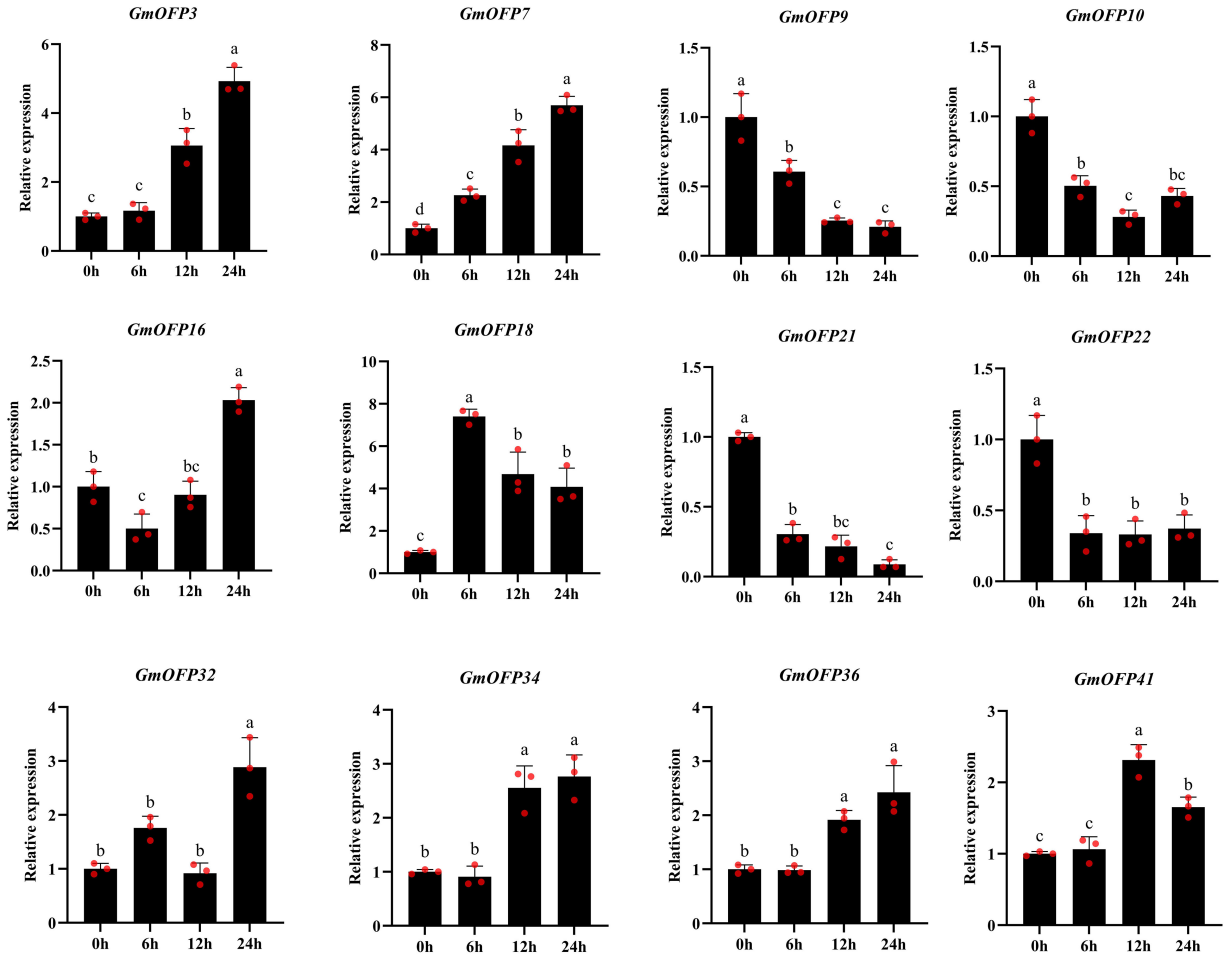
Relative expression levels of 12 *GmOFP* genes in soybean. The sample size was n = 3. The data are presented as the means ± SDs. Different lowercase letters indicate significant differences within the same treatment at different times (P < 0.05).

To investigate the expression patterns of soybean *OFP* gene family members under salt stress, we randomly selected 12 genes from 42 *GmOFPs* to validate the data in the transcriptome. we performed qPCR validation on a subset of salt-responsive genes identified from soybean salt stress transcriptomic data. Our analysis revealed differential responsiveness to salt stress across all 12 examined *GmOFP* genes (**Figure 9**). Comparative analysis with untreated controls (0-hour treatment) revealed temporally specific expression dynamics: At 6 hours post-treatment, *GmOFP7* and *GmOFP18* presented rapid salt stress induction with significant upregulation, whereas *GmOFP9*, *GmOFP10*, *GmOFP16*, *GmOFP21*, and *GmOFP22* presented marked downregulation. No significant expression changes were detected in *GmOFP3*, *GmOFP32*, *GmOFP34*, *GmOFP36*, or *GmOFP41* at this timepoint. After 12 hours of salt exposure, *GmOFP3*, *GmOFP7*, *GmOFP18*, *GmOFP34*, *GmOFP36*, and *GmOFP41* were significantly upregulated, whereas *GmOFP9*, *GmOFP10*, *GmOFP21*, and *GmOFP22* were markedly downregulated. *GmOFP16* and *GmOFP32* maintained baseline expression levels during this phase. After 24 hours of treatment, eight genes (*GmOFP3*, *GmOFP7, GmOFP16, GmOFP18, GmOFP32, GmOFP34*, *GmOFP36*, and *GmOFP41*) were significantly upregulated, whereas four members (*GmOFP9, GmOFP10, GmOFP21*, and *GmOFP22*) remained downregulated. Notably, the qPCR results showed substantial concordance with the transcriptomic data for 11 genes, with *GmOFP32* being the sole exception. This validation confirms the reliability of transcriptome-based preliminary screening for identifying salt-responsive *GmOFP* genes and provides a valuable reference for subsequent mechanistic investigations into soybean salt stress adaptation.

## 4 Discussion

The *OFP* family, as a plant-specific transcription factor family, have been extensively studied and characterized in various species, demonstrating their crucial roles in plant growth and development (Wang et al., 2010; Zhang et al., 2016; Liu et al., 2022; Wang et al., 2020b). The *OFP* gene family of soybean, a globally important economic crop and vital source of dietary protein, remains underexplored. Previous studies identified 19 *AtOFPs* in *Arabidopsis* (Liu et al., 2014), 31 *OsOFPs* in rice (Yu et al., 2015), 31 *SlOFPs* in tomato (Huang et al., 2013), and, remarkably, 100 *TaOFPs* in hexaploid wheat (*Triticum aestivum L.*), revealing significant family expansion in polyploid species (Wang et al., 2020a). Given that the complex paleopolyploid background of soybean is shaped by two whole-genome duplication events, our study identified 42 *GmOFP* members in the soybean genome. This number exceeds that of the diploid model plant *Arabidopsis* but remains lower than the 56 members reported in allotetraploid upland cotton (*Gossypium hirsutum*) (Zhang et al., 2022). Such quantitative differences likely reflect species-specific evolutionary trajectories involving gene duplication events and functional diversification during genome evolution.

During the structural analysis of the soybean *GmOFP* genes, we observed that most family members contained only a single exon (**Figure 2**). This characteristic single-exon architecture devoid of introns is highly conserved with *OFP* family members in *Arabidopsis*, rice, and other species (Liu et al., 2014; Yu et al., 2015), suggesting potential evolutionary maintenance of structural simplicity through functional selection. Single-exon genes demonstrate accelerated transcriptional activation in response to abiotic stresses, a phenomenon likely attributable to the absence of splicing regulation steps that could otherwise delay transcriptional initiation (Hu et al., 2023; Jorquera et al., 2018). Chromosomal distribution analysis revealed the preferential localization of *GmOFPs* in subtelomeric regions (**Figure 3**), which was potentially associated with the unique epigenetic regulatory milieu of the chromosomal termini. These telomere-proximal domains typically maintain open chromatin configurations, which may facilitate transcription factor accessibility and rapid transcriptional responses to environmental signals (Philimonenko et al., 2010).

*OFPs* play diverse roles across various growth and developmental processes. Molecular mechanistic studies in peach (*Prunus persica*) revealed that PpOFP4 and PpOFP5 exhibit self-assembly properties to form homodimers. Notably, PpOFP5 specifically binds with either PpOFP7 or PpOFP8 to form heterodimeric complexes (Li et al., 2019b). This multilayered protein interaction network potentially operates through dual regulatory pathways: it may modulate either the transcriptional activation potency of the proteins themselves or their DNA-binding efficiency, thereby collectively orchestrating the expression regulation of downstream target genes. Parallel investigations on the *OFP* family in apple (*Malus pumila Mill.*) indicated that MdOFP13, MdOFP16, MdOFP20, and MdOFP2 can assemble into heterodimers with other MdOFP members, while MdOFP16 additionally possesses a self-association capacity to form homodimers (Li et al., 2019a). In our study, based on tissue-specific expression pattern analysis and phylogenetic analysis, we speculate that analogous dimers may also occur in soybean. This possibility is suggested by the co-high expression of specific *GmOFP* members within distinct tissues, such as *GmOFP8, GmOFP18, GmOFP27*, and *GmOFP28* in the shoot apical meristem and *GmOFP14* and *GmOFP33* in the roots (**Figure 7**). However, whether GmOFP proteins possess similar interaction networks remains to be experimentally validated and requires further investigation.

With respect to the regulation of plant fruit morphology, researchers have reported that *CmFSI8/CmOFP13*, a member of the *OFP* family in melon (*Cucumis melo*), has a potent inhibitory effect on organ elongation growth when overexpressed in the model plant *Arabidopsis* (Ma et al., 2022). This gene has ovary-specific expression patterns during reproductive development in melon, with genomic variations in its promoter region critically influencing transcriptional activity and function determination. In this study, our expression profiling revealed that a substantial number of *GmOFP* genes are expressed in soybean flowers (**Figure 7**). However, whether these genes play similar roles in floral organ morphogenesis remains unknown.

In previous studies, researchers focused primarily on the roles of *OFPs* in plant organogenesis and fruit morphological development. However, accumulating evidence has recently revealed their critical involvement in abiotic stress responses. In rice, multiple *OsOFPs* display responsive expression patterns under salt and drought stress conditions, suggesting their potential functional significance in mitigating these abiotic stresses (Ahmad et al., 2023). Furthermore, in rice, *OsOFP6* not only plays essential roles in diverse growth stages and developmental processes but also acts as a positive regulator of drought and cold stress adaptation (Ma et al., 2017). Phylogenetic analysis revealed that specific *GmOFPs* in soybean (e.g., *GmOFP5, GmOFP9*, and *GmOFP22*) clustered within the same clade as *OsOFP6* (**Figure 1**), indicating a close phylogenetic relationship. This high degree of homology suggests that these *GmOFPs* may share functional similarity with *OsOFP6*, although further experimental validation is needed. In this study, we also investigated the roles of soybean *GmOFPs* in the response to abiotic stress. Through systematic mining and screening of soybean transcriptomic data under salt stress combined with qPCR validation, we identified a substantial number of *GmOFPs* that presented significant alterations in relative expression levels under saline conditions. These findings imply that these *GmOFPs* may have crucial biological functions in mediating salt stress adaptation during soybean growth and development.

## 5 Conclusions

In this study, we conducted a comprehensive genome-wide identification and bioinformatics analysis of the *OFP* gene family in soybean. A total of 42 *GmOFPs* were systematically identified and found to be unevenly distributed across 19 soybean chromosomes. Notably, most members of this family are characterized as single-exon genes. Through integrated gene expression profiling and experimental validation, we revealed that *GmOFPs* exhibit varying expression levels across different soybean tissues. Furthermore, several *GmOFPs* demonstrated differential responsiveness to salt stress conditions, suggesting their potential functional involvement in regulating soybean growth and developmental processes under salinity challenges. These findings establish a crucial theoretical foundation for subsequent investigations into the molecular mechanisms underlying *OFP*-mediated stress adaptation in soybean.

## Data Availability

The original contributions presented in the study are included in the article/**Supplementary Material**. Further inquiries can be directed to the corresponding authors.

## Supplementary material

Supplementary Table 1 Primer sequences used for qPCR.

Supplementary Table 2 Information on soybean *OFP* family members.

Supplementary Figure 1 Conserved Motif-Logo graphs.

Supplementary Figure 2 Multiple sequence alignment.
