# Supplementary material for: Genome-wide identification of the OVATE gene family of proteins in soybean and expression profiling under salt stress
Source: Front Plant Sci. 2025 Sep 8;16:1682513. doi: 10.3389/fpls.2025.1682513 (PMC12451945; doi:10.3389/fpls.2025.1682513)
Supplement: Supplementary Table 1 — Primer sequences used for qPCR. [file SupplementaryFile1.docx]

Supplementary Material

# Supplementary Tables

**1.1 Table S1. Primer sequences used for qPCR**

| **Primer Name** | **Sequence (5’-3’)** |
| --- | --- |
| *GmOFP3-qF* | GCCTCCCAACGCTTCTTCTTC |
| *GmOFP3-qR* | TCTTCTCTCAACAATGGTGGTAAGTG |
| *GmOFP7-qF* | CCCCATTTGACACCTCTCCC |
| *GmOFP7-qR* | ATGGTGTCGTTGGTGGTGTT |
| *GmOFP9-qF* | GTTGGCGCTTTCGTCGATTT |
| *GmOFP9-qR* | CGAGTGCTGCAAGAGGAAGA |
| *GmOFP10-qF* | ACCAACACCAACACCACATTCTC |
| *GmOFP10-qR* | CTCCACAGCCACACCTTCTTTC |
| *GmOFP16-qF* | TTGTAATGGTAGCAATGGAGAAGAGC |
| *GmOFP16-qR* | GGCGTCTTGAAGGCGGTTTG |
| *GmOFP18-qF* | ACAGATCAGCGAGCCAGAAG |
| *GmOFP18-qR* | TACACGAACTGGCTTGGCTC |
| *GmOFP21-qF* | TCTACCTTCTTCGAGCCCGA |
| *GmOFP21-qR* | TCAACAATGCTGGGTGTCGA |
| *GmOFP22-qF* | ACTCTCATTCTCGTCCTTCTTCTCC |
| *GmOFP22-qR* | AGCACCAACCTCTTCCTCTTCC |
| *GmOFP32-qF* | AATGCTGGAGATGATTGTGGAGAAC |
| *GmOFP32-qR* | TCACTTGAATTGAGGGAAAGGTAGC |
| *GmOFP34-qF* | AACAGGCAGAAGCAACCACATC |
| *GmOFP34-qR* | TGGAGAATGTATTTTGACCCTAGAGC |
| *GmOFP36-qF* | TCAGGGAGTCAATGGTGGAGATG |
| *GmOFP36-qR* | AGAGATAGCAGGCAAGCAAGTTC |
| *GmOFP41-qF* | GCTGCTTCTAATGGTTCTAACACTTG |
| *GmOFP41-qR* | TTCCCAATCCCATGCTCAACAATC |
| *GmCYP2-qF* | CGGGACCAGTGTGCTTCTTCA |
| *GmCYP2-qR* | CCCCTCCACTACAAAGGCTCG |

**1.2 Table S2. Information on soybean *OFP* family members**

| **Sequence ID** | **Gene ID** | **Number of Amino Acid** | **Molecular Weight** | **Theoretical pI** | **Instability Index** | **Aliphatic Index** | **Grand Average of Hydropathicity** |
| --- | --- | --- | --- | --- | --- | --- | --- |
| *GmOFP1* | Glyma.01G131900 | 385 | 43913.46 | 9.91 | 55.93 | 60 | -0.954 |
| *GmOFP2* | Glyma.01G200600 | 166 | 19254.02 | 4.34 | 38.94 | 95.12 | 0.28 |
| *GmOFP3* | Glyma.02G146400 | 204 | 22940 | 5.85 | 51.46 | 71.23 | -0.339 |
| *GmOFP4* | Glyma.02G199300 | 174 | 19493.85 | 9.18 | 57.29 | 67.82 | -0.468 |
| *GmOFP5* | Glyma.02G199500 | 276 | 30825.68 | 4.55 | 67.01 | 59.67 | -0.604 |
| *GmOFP6* | Glyma.02G235600 | 383 | 43196.32 | 8.74 | 71.86 | 63.16 | -0.699 |
| *GmOFP7* | Glyma.03G036600 | 387 | 44107.54 | 9.97 | 57.31 | 56.9 | -1.014 |
| *GmOFP8* | Glyma.03G155600 | 250 | 27750.74 | 5.82 | 53.62 | 56.24 | -0.693 |
| *GmOFP9* | Glyma.03G200100 | 283 | 30843.11 | 5.04 | 74.28 | 63.32 | -0.458 |
| *GmOFP10* | Glyma.03G200200 | 190 | 21286.89 | 9.64 | 49.08 | 65.63 | -0.624 |
| *GmOFP11* | Glyma.04G033000 | 259 | 29804.28 | 9.71 | 76.02 | 58.69 | -0.733 |
| *GmOFP12* | Glyma.05G162200 | 409 | 46922.12 | 9.32 | 61.96 | 61.49 | -0.986 |
| *GmOFP13* | Glyma.05G230900 | 285 | 33007.41 | 9.73 | 65.32 | 60.49 | -0.823 |
| *GmOFP14* | Glyma.06G033000 | 252 | 29022.59 | 9.57 | 76.24 | 64.92 | -0.644 |
| *GmOFP15* | Glyma.07G006200 | 282 | 32495.92 | 8.61 | 57.86 | 67.06 | -0.71 |
| *GmOFP16* | Glyma.07G263900 | 156 | 18212.6 | 7.63 | 69.66 | 62.5 | -0.667 |
| *GmOFP17* | Glyma.08G038400 | 293 | 33970.59 | 9.82 | 63.96 | 60.17 | -0.842 |
| *GmOFP18* | Glyma.08G119800 | 413 | 47663.06 | 8.97 | 68.14 | 61.6 | -0.975 |
| *GmOFP19* | Glyma.08G206800 | 188 | 21862.26 | 9.93 | 57.18 | 75.64 | -0.649 |
| *GmOFP20* | Glyma.09G276400 | 386 | 43915.27 | 9.76 | 53.01 | 60.88 | -1 |
| *GmOFP21* | Glyma.10G027500 | 219 | 24720.88 | 5.98 | 54.47 | 67.72 | -0.449 |
| *GmOFP22* | Glyma.10G077700 | 267 | 29764.72 | 4.57 | 62.56 | 67.87 | -0.494 |
| *GmOFP23* | Glyma.10G077800 | 177 | 19921.44 | 9.35 | 51.89 | 67.23 | -0.507 |
| *GmOFP24* | Glyma.10G247600 | 180 | 20293.98 | 8.75 | 52.79 | 74.72 | -0.356 |
| *GmOFP25* | Glyma.10G277300 | 251 | 27964.76 | 5.17 | 42.02 | 57.85 | -0.595 |
| *GmOFP26* | Glyma.11G159800 | 197 | 22126.39 | 6.09 | 76.67 | 61.32 | -0.841 |
| *GmOFP27* | Glyma.11G180300 | 385 | 43690.46 | 9.49 | 72.45 | 65.32 | -0.694 |
| *GmOFP28* | Glyma.12G093200 | 195 | 21928.38 | 6.45 | 70.54 | 64.41 | -0.798 |
| *GmOFP29* | Glyma.12G187500 | 186 | 21010.6 | 8.19 | 43.55 | 56.61 | -0.935 |
| *GmOFP30* | Glyma.13G225800 | 414 | 47883.35 | 9.12 | 64.35 | 63.57 | -1.014 |
| *GmOFP31* | Glyma.13G314000 | 184 | 20772.27 | 7.66 | 61.01 | 60.92 | -0.874 |
| *GmOFP32* | Glyma.14G203600 | 374 | 42438.78 | 8.82 | 74.25 | 68.58 | -0.665 |
| *GmOFP33* | Glyma.15G014500 | 249 | 28473.75 | 6.76 | 36.69 | 67.71 | -0.721 |
| *GmOFP34* | Glyma.15G086302 | 171 | 20148.25 | 8.95 | 65.59 | 71.81 | -0.917 |
| *GmOFP35* | Glyma.17G010000 | 152 | 17758.17 | 6.99 | 60.57 | 60.92 | -0.592 |
| *GmOFP36* | Glyma.18G054200 | 385 | 43585.24 | 9.31 | 75.54 | 68.1 | -0.655 |
| *GmOFP37* | Glyma.18G215300 | 377 | 43275.8 | 9.79 | 51.8 | 62.04 | -0.972 |
| *GmOFP38* | Glyma.19G157700 | 262 | 28570.96 | 5.41 | 63.19 | 66.76 | -0.448 |
| *GmOFP39* | Glyma.19G197700 | 272 | 29871.04 | 4.74 | 64 | 67.02 | -0.317 |
| *GmOFP40* | Glyma.19G197800 | 224 | 25018.26 | 9.15 | 44.26 | 65.67 | -0.524 |
| *GmOFP41* | Glyma.20G112400 | 239 | 26446.24 | 4.84 | 39.36 | 58.33 | -0.47 |
| *GmOFP42* | Glyma.20G147700 | 190 | 21516.49 | 8.75 | 55.82 | 77.47 | -0.282 |

# Supplementary Figures

## Figure S1. Conserved Motif-Logo graphs


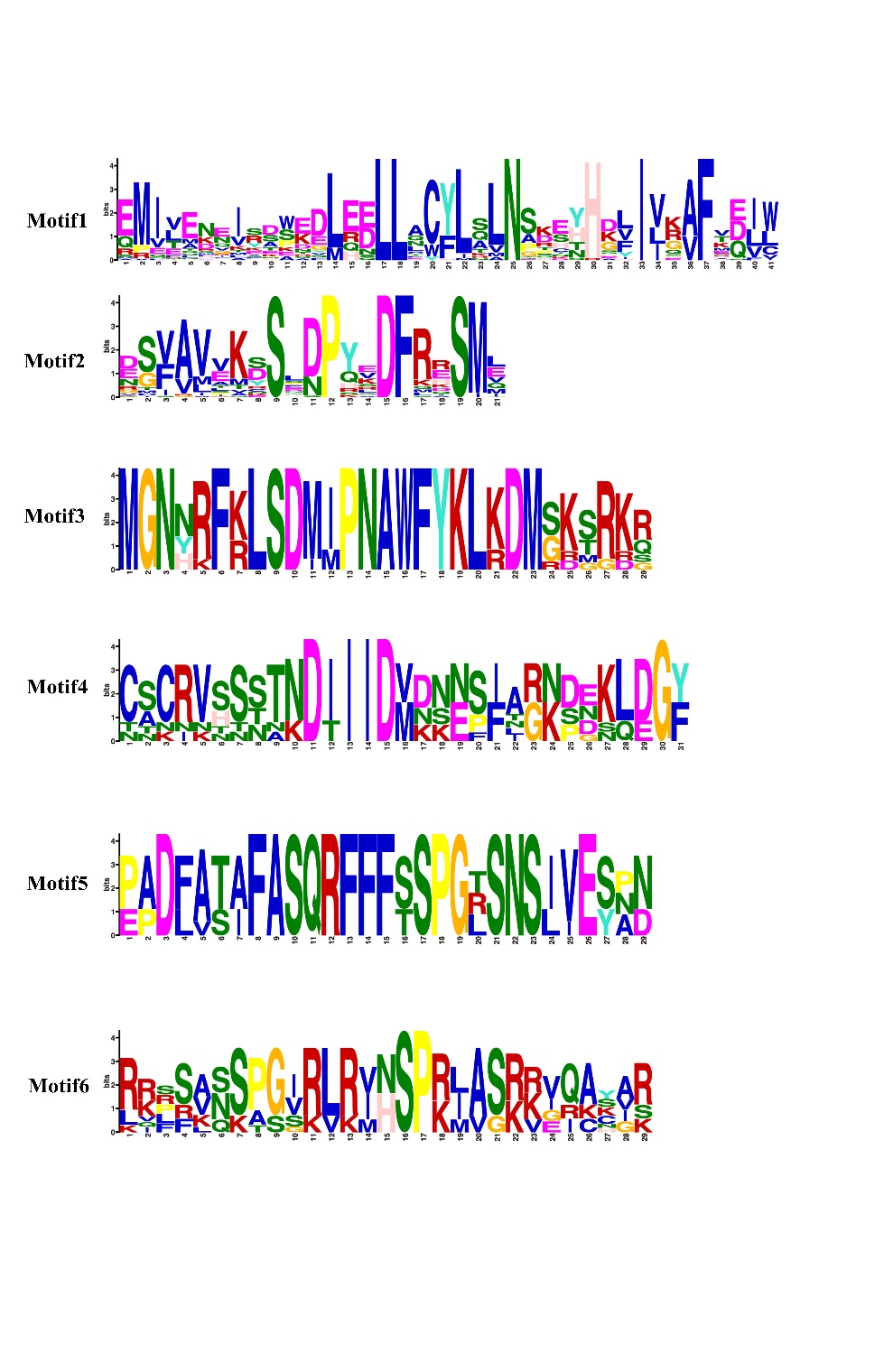


Figure S1． Conserved Motif-Logo graphs

## Figure S2. Multiple sequence alignment


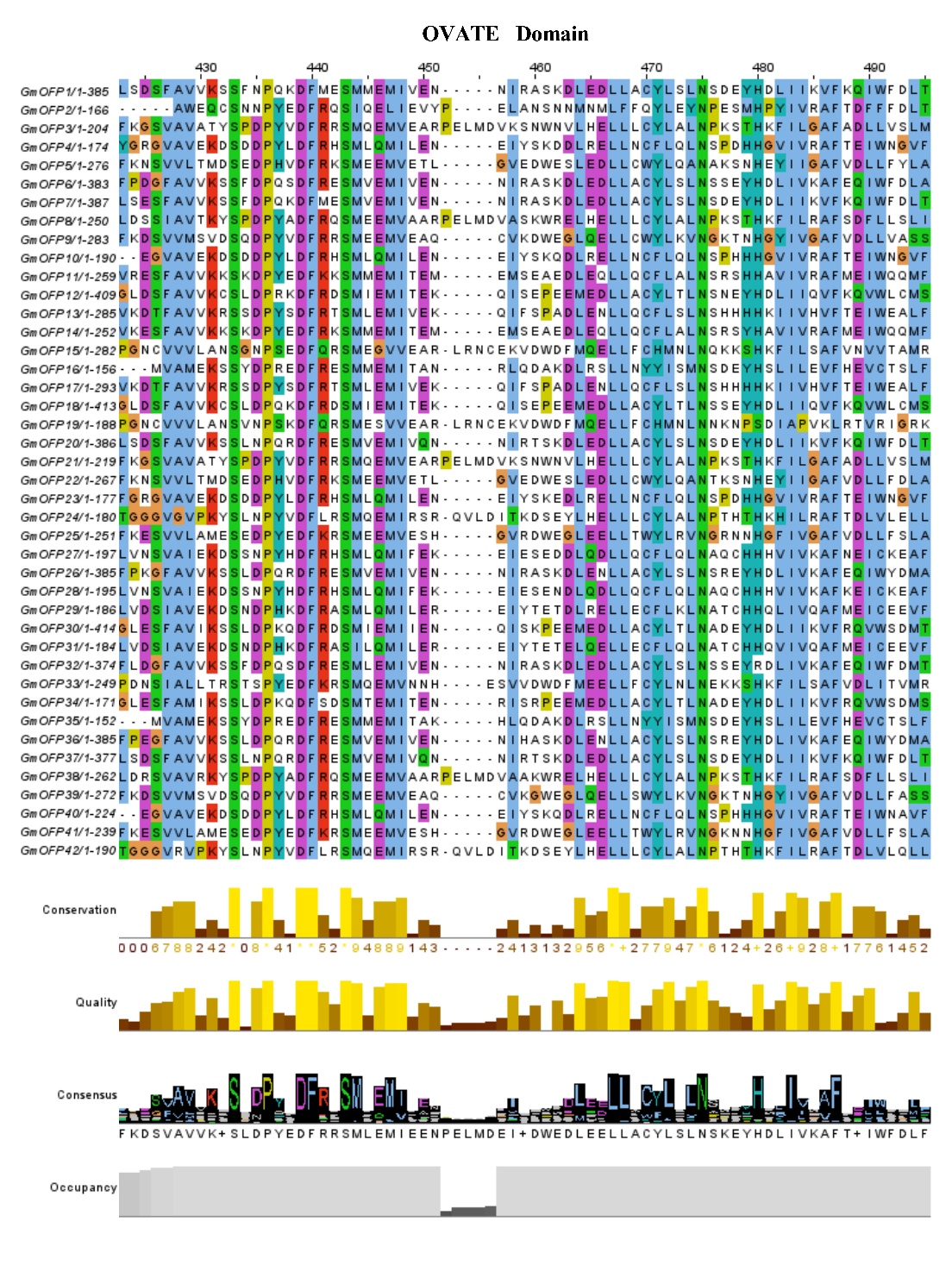


Figure S2. Multiple sequence alignment
